# CD105 overexpression mediates drug-resistance in choriocarcinoma cells through BMP9/Smad pathway

**DOI:** 10.7150/jca.34965

**Published:** 2020-01-01

**Authors:** Xiaoyu Wang, Liju Zong, Wenze Wang, Junjun Yang, Yang Xiang

**Affiliations:** 1 Department of Obstetrics and Gynecology, Peking Union Medical College Hospital, Chinese Academy of Medical Sciences and Peking Union Medical College, No.1 Shuaifuyuan, Dongcheng District, Beijing 100730, China.; 2 Department of Pathology, Peking Union Medical College Hospital, Chinese Academy of Medical Sciences and Peking Union Medical College, No.1 Shuaifuyuan, Dongcheng District, Beijing 100730, China.

**Keywords:** BMP9/Smad pathway, CD105, chemoresistance, choriocarcinoma

## Abstract

**Background:** CD105 (endoglin, ENG) is a membranous protein that is overexpressed in tumor-associated endothelial cells and some actual tumor cells and is associated with poor prognosis. However, the association between CD105 and response to chemoresistance in choriocarcinoma cells has not been clearly defined. The present study aimed to investigate the effects of targeting CD105 in drug-resistant choriocarcinoma.

**Methods:** CD105 expression was evaluated in drug-resistant and parental choriocarcinoma cells by qRT-PCR, western blotting, and immunofluorescence. CD105 overexpressing and knockdown cells were established by lentiviral transfection. CCK8, transwell, and flow cytometric assays were used to measure changes in drug-sensitivity, invasion, migration, and apoptosis. Drug-sensitivity and Smad1/5/8, Smad2, and Smad3 expression were also detected after BMP9 treatment. Immunohistochemical staining for CD105 and BMP9 was performed on choriocarcinoma tissues and the relationships between clinical and pathological characteristics were analysed.

**Results:** Data demonstrated that CD105 overexpression could decrease drug sensitivity, promote invasion and migration, and inhibit apoptosis in choriocarcinoma cells, and this protein was confirmed to mediate drug resistance through the BMP9/Smad pathway. Further experiments showed that the expression of CD105 and BMP9 was consistent in choriocarcinoma tissues and significantly associated with disease recurrence.

**Conclusions:** This study provides evidence suggesting that CD105 is critical for the development of drug-resistance in choriocarcinoma and might serve as a therapeutic target for reversing chemoresistance in choriocarcinoma patients.

## Introduction

Choriocarcinoma is a highly aggressive tumor that usually occurs in women of child-bearing age. Because of the application of useful chemotherapeutic drugs, the overall cure rate of this disease is higher than 90% and the majority of patients that achieve complete remission rely only on chemotherapy. However, 20% of patients with recurrent disease will die from a lack of available effective therapies, making drug resistance the main clinical challenge in this disease 1. This emphasizes the necessity to develop new approaches for the treatment of these patients.

CD105 is a 180-kDa disulphide-linked homodimeric transmembrane protein that functions as a component of the transforming growth factor-β (TGF-β) receptor complex. As a well-characterized angiogenic marker, it is highly expressed on proliferating endothelial cells and has good specificity for the assessment of tumor angiogenesis. Recently, anti-angiogenic agents have received extensive attention as new therapeutic modalities, and CD105 has become an additional target through which intratumoral angiogenesis might be targeted 2. However, CD105 might function in a capacity beyond angiogenesis alone. Studies on solid tumors suggest that it is upregulated not only in tumor endothelial cells, but also in actual tumor cells, and is associated with poor prognosis. CD105 silencing was shown to reduce the proliferation, survival, and migration of melanoma cells; thus this was found to have anti-tumor effectiveness 3. In early breast cancer, patients with high stromal CD105 expression had shorter disease-free survival, metastasis-free survival, and overall survival than patients with negative/low CD105 expression 4. In gastrointestinal stromal tumors, strong CD105 staining was also found to be associated with malignant and high-risk tumors. Bone morphogenetic proteins (BMPs) belong to the TGF-β superfamily and consist of at least 20 members in humans. BMP9 is responsible for more than 60% of the activity of BMPs and can directly interact with the hydrophobic surface of the N-terminal orphan domain of CD105 5. However, no relevant study regarding BMP9 has been performed for choriocarcinoma.

In a recent study, a patient with metastatic and refractory choriocarcinoma following a combination of chemotherapy and surgical metastectomy achieved complete remission after receiving therapy with an anti-CD105 monoclonal antibody (TRC105) and bevacizumab, which indicated that CD105 is a potential novel drug target for overcoming chemoresistance in choriocarcinoma 6. However, the mechanism through which CD105 affects chemoresistance in choriocarcinoma cells has not been clearly defined. This study was undertaken to provide insight regarding the molecular mechanism associated with drug-resistant choriocarcinoma and to serve as a reference for future targeted treatment regimens for chemoresistant choriocarcinoma patients. As such, we focused on delineating the role of CD105 in this disease, as well as the underlying mechanism.

## Methods and Materials

### Cell lines and reagent

The human placental choriocarcinoma JEG-3 cell line was obtained from the cell culture centre of the Basic Medicine Institution (BMI) of Peking Union Medical College (Beijing, China). It was cultured in high glucose Dulbecco's modified Eagle's medium (DMEM; Gibco, Thermo Fisher Scientific, USA), supplemented with 10% fetal bovine serum (FBS; Gibco), 50 U/mL penicillin (Solarbio, Beijing, China), and 50 U/mL gentamicin (Solarbio) in a 5% CO_2_ humidified incubator at 37ºC. The human methotrexate (MTX) -resistant choriocarcinoma JEG-3 (JEG-3/MR) cell line and floxuridine (FUDR) -resistant choriocarcinoma JEG-3 cell line (JEG-3/FR) used in the present study were previously established and described in our published study 7, 8. Recombinant human BMP-9 protein was purchased from R&D Systems (3209-BP, Bio-Techne Corporation, USA), and MTX and FUDR were purchased from Sigma-Aldrich (USA).

### Patient samples and tissue collection

Thirty-three choriocarcinoma samples were collected by hysterectomy from 2010 to 2015 at Peking Union Medical College Hospital. Patients with confirmed choriocarcinoma based on histology with no medical history of prior gestational trophoblastic disease, chemo-resistant lesions after combination chemotherapy, and complete follow-up data available were included in this study. The prior chemotherapy regimens included FAV (FUDR, dactinomycin, vincristine), FAEV (FUDR, dactinomycin, etoposide, vincristine) and EMA/CO (etoposide, MTX, dactinomycin/cyclophosphamide, vincristine). Drug-resistance was determined that after 2 cycles of combination chemotherapy, the β-hCG did not decline logarithmically, remained at a plateau or increased level. The indication of hysterectomy was the diameter of uterine lesion larger than 3cm in the drug-resistant patients. Regarding the use of these tissues for research purposes, prior consent was obtained from the patients.

### Lentiviral transfection

The lentiviral vector for CD105 gene overexpression was achieved by recombining pLenti-EF1a-EGFP-P2A-Puro-CMV -3Flag vector with the *Eng* gene; while the lentivirus vector for CD105 knockdown was achieved by cloning small hairpin RNAs (shRNAs) using a self-inactivating lentivirus vector containing a CMV-driven GFP reporter and a U6 promoter. All the recombining and negative control viruses carried the green fluorescent protein (GFP) gene and constructed by Obio Technology Corp., Ltd. (Obio, Shanghai, China). JEG-3 cells in the logarithmic growth phase were seeded into 96-well plates. Following 12 h of culture, the supernatant was discarded and 100 µl/well of diluted virus suspension was added to medium. After overnight culture, the transfection mixture was replaced with normal complete growth medium to avoid cell toxicity. After 48 h, transfection efficiency was monitored using fluorescence microscopy, and each single cell was seeded into 96-well plate in the culture of the medium with puromycin (0.5 µg/ml). The culture medium was replaced every 2 days to remove dead cells. The transfection efficiency was observed by fluorescence microscopy and confirmed by qPCR and western blot analyses.

### Quantitative RT-PCR (qRT-PCR)

Total RNA was extracted using TRIZOL (Invitrogen, Thermo Fisher Scientific, Inc.). cDNA was synthesized using 2 µg of total RNA using the PrimeScript™ RT reagent Kit with gDNA Eraser (Takara Biotechnology Co., Ltd. Dalian, China). qRT-PCR analysis was performed using SYBR^®^ Premix Ex Taq™ II (Perfect Real time; Takara) and under thermal cycling parameters of 95 °C for 30 sec followed by 40 cycles of 3 sec at 95 ^o^C and 40 sec at 60 °C with the 7500 Fast Real-Time PCR System (Applied Biosystems, Thermo Fisher Scientific, Inc.). The primers for CD105 (forward: 5′ CGCACCGATCCAGACCACTC 3′; reverse: 5′ CCCGGCTCGATGGTGTTGGA 3′), BMP9 (forward: 5′ CTGCCCTTCTTTGTTGTCTT 3′; reverse: 5′ CCTTACACTCGTAGGCTTCATA 3′) and GAPDH (forward: 5′ CAGCGACACCCACTCCTC 3′; reverse: 5′ TGAGGTCCACCACCCTGT 3′) were constructed by TsingKe Bio-Technology Co., Ltd. (Beijing, China). Each sample was assayed in triplicate and the data were analysed using the 2^-ΔΔCq^ method.

### Western blotting

Whole cell lysates and western blotting were performed as described previously 9. Antibodies against CD105 (ab169545, 1:1000), Smad2 (ab40855, 1:2000), pSmad3 (ab52903, 1:1000) were purchased from Abcam. An antibody against BMP9 (sc514211, 1:500) was purchased from Santa Cruz Biotechnology (USA). Antibodies against Smad1 (6944, 1:1000), pSmad1/5/8 (13820, 1:1000), and pSmad2 (3108, 1:1000) were purchased from Cell Signaling Technology. Antibodies against Smad3 (YM3417, 1:2000), GAPDH (YM3029, 1:20000), and β-tubulin (YM3030, 1:5000) were purchased from ImmunoWay Biotechnology.

### Proliferation assays

Cells were seeded at a density of 2000 cells/well in 96-well plates. Following 12 h of culture, drugs were added into medium and the cells were incubated for another 48 h at 37 °C. At the end of the experiment, 10 μL of CCK-8 solution (Dojindo, Japan) was used to assess cell growth by measuring the absorbance at a wavelength of 450 nm using a Varioskan Flash microplate reader (Thermo). All data were obtained from three independent experiments. The half-maximal inhibitory concentration values (IC_50_) were estimated from CCK8 assays using probit analysis.

### Invasion and migration assay

Invasion and migration assays were performed using a 24-well Transwell chamber (Corning, USA). The chamber was coated with 20 μL of Matrigel (BD Bioscience, USA) at a dilution of 1:4 for invasion assays. Cells were resuspended in DMEM without FBS and 200 μL (1 × 10^5^/mL) of cell suspensions were seeded in the top chamber. The lower compartment contained 500 μL of DMEM supplemented with 20% FBS. After incubation for 24 h for migration or 48 h for invasion assays, the migrated or invaded cells were fixed with formaldehyde and stained with crystal violet. These cells were then quantified under a microscope based on six random fields.

### Flow cytometric analysis

Apoptosis was assessed by flow cytometry using a BD Accuri C6 (BD Biosciences). The Annexin V-PE/7AAD Kit (BD Bioscience) was used for analysing apoptosis and performed in accordance with manufacturer's instructions. Data were analysed using the FlowJo V7 software (Tree Star, OR, USA). Experiments were performed independently in triplicate for this study.

### Immunofluorescence (IF)

Cells were fixed with 4% paraformaldehyde for 20 min, permeabilized with 0.3% Triton X-100 for 10 min, and blocked with 5% BSA for 2 h at room temperature. Then, the cells were incubated with primary antibodies including mouse anti-CD105 antibody (Abcam, ab11414, 1:500) and rabbit anti-BMP9 antibody (Abcam, ab35088, 1:300) overnight at 4 °C. The next day, the cells were incubated with corresponding secondary antibodies including Alexa Fluor 488-labelled goat anti-mouse (green, Servicebio, GB2530, 1:400), AMCA goat anti-mouse (blue, Servicebio, GB26301, 1:50), and CY3 goat anti-rabbit (red, Servicebio, GB21303, 1:300) for 1 h; nuclei were stained with DAPI (Servicebio) for 5 min at room temperature. Before each step, cells were washed three times with PBS. The stained cells were mounted with antifade mounting medium, and images were acquired under a confocal fluorescence microscope.

### HE staining and immunohistochemistry (IHC)

HE staining and IHC analysis for CD105 and BMP9 in choriocarcinoma cells were performed for samples from patients who received hysterectomy during chemotherapy. The procedures were performed following in accordance with a previous report 10. The following primary antibodies were used: rabbit monoclonal anti-human CD105 (Abcam, ab169545, 1:900) and rabbit polyclonal anti-human BMP9 (Abcam, ab35088, 1:100). The staining results were graded as 3 to 0. When > 90% or 90-50% of tumor cells showed moderate/strong staining, the grade was 3 or 2. Grade 1 denoted cells with moderate/strong staining in less than 50% of tumor cells or with weak staining. The grade was 0 when tumor cells with no staining.

### Statistical analysis

All statistical analyses were performed with SPSS 22.0 (SPSS, Chicago, IL, USA). The means of normally distributed continuous data between two groups were analysed by performing Student's t-tests. For IHC analysis, the Mann-Whitney U test was applied to compare the expression intensity. The correlation between CD105 and BMP9 expression was analysed by a Spearman test. A value of P < 0.05 was considered statistically significant.

## Results

### CD105 is up-regulated in drug-resistant choriocarcinoma cells

**Figure 1:**
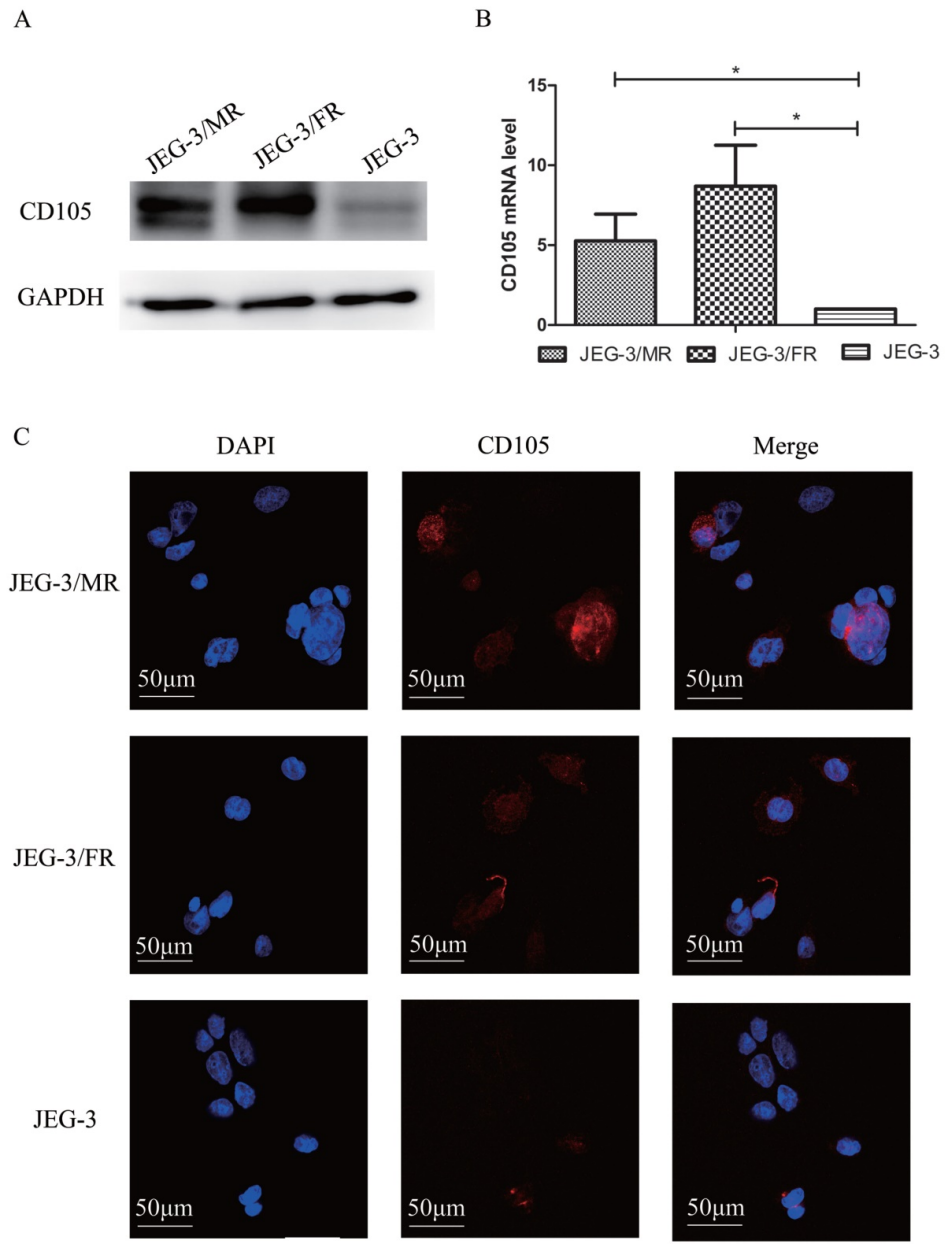
** Expression of CD105 in human placental choriocarcinoma cell line (JEG-3), MTX-resistant choricarcinoma cell line (JEG-3/MR) and FUDR-resistant choricarcinoma cell line (JEG-3/FR).** Western blotting (A), qRT-PCR (B) and immunofluorescence (C) analysis confirmed the overexpression of CD105 in JEG-3/MR and JEG-3/FR cells. The image C is at ×600 magnification.

To investigate the potential role of CD105 in drug-resistant choriocarcinoma, we determined the expression of CD105 by western blotting, qRT-PCT and IF in JEG-3, JEG-3/FR and JEG-3/MR cells (Fig. 1). Western blotting and qRT-PCR results showed that CD105 was up-regulated in JEG-3/FR and JEG-3/MR cells compared to expression in JEG-3 cells. By IF, a cell membrane location of CD105 was observed in the three cell lines. The fluorescence intensity in JEG-3/FR and JEG-3/MR cells was higher than that in JEG-3 cells. All results suggested that CD105 expression is increased in JEG-3/FR and JEG-3/MR cells.

### CD105 decreases drug-sensitivity in JEG-3 cells

**Figure 2:**
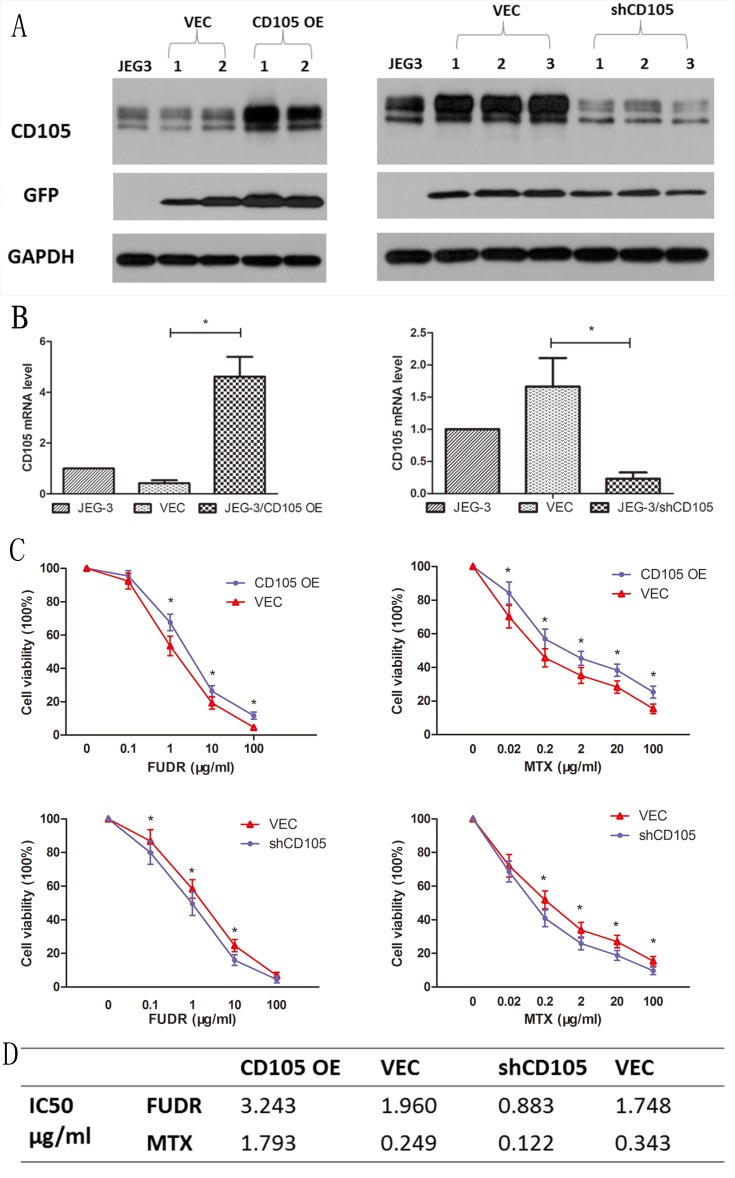
** CD105 decreases drug-sensitivity in JEG-3 cells.** Western blot (A) and qRT-PCR (B) confirmed the overexpression or knockdown of CD105 in stable monoclonal CD105-transfected (JEG-3/CD105 OE) or shRNA-transfected (JEG-3/shCD105) cell. (C) Cell viability by a CCK8 assay after treating with different concentrations of MTX or FUDR (*, P<0.05, Student's t-test). (D) IC_50_ values for MTX and FUDR with respect to JEG-3/CD105 OE and JEG-3/shCD105 cells indicated that overexpression of CD105 significantly decreased the sensitivity, whereas knockdown of CD105 increased drug sensitivity.

Lentiviral vectors were used to establish CD105-overexpressing (JEG-3/CD105 OE) and CD105-knockdown (JEG-3/shCD105) cells, as well as respective vector control lines. Results of qRT-PCR and western blotting in different single cell clones after lentiviral transfection revealed that the expression of CD105 in JEG-3/CD105 OE cells was significantly increased compared to that in the vector and control groups, whereas in JEG-3/shCD105 cells, this was significantly decreased (Fig. 2A and B).

To verify the effect of CD105 on drug-sensitivity in JEG-3 cells, we performed CCK-8 assays to measure cell viability after treating the cells with different concentrations of MTX or FUDR. Results indicated that overexpression of CD105 significantly decreased the sensitivity of JEG-3 cells to MTX and FUDR, whereas knockdown of CD105 increased drug sensitivity (Fig. 2C). The IC_50_ values for MTX and FUDR with respect to JEG-3/CD105 OE and JEG-3/shCD105 cells and corresponding vector control cells are listed in Fig 2D.

### CD105 promotes migration and invasion, and inhibits apoptosis in JEG-3 cells

**Figure 3:**
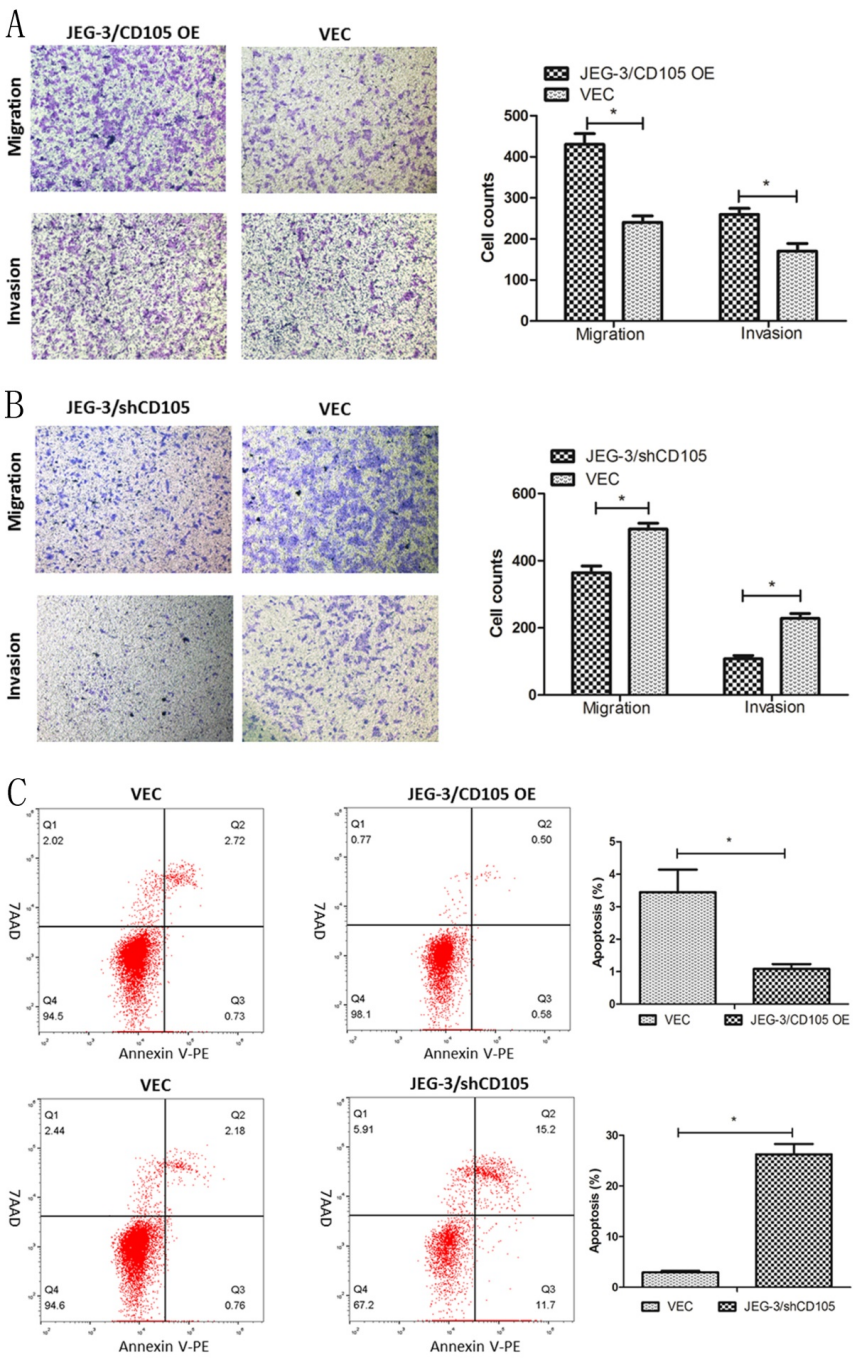
** CD105 promotes migration and invasion, and inhibits apoptosis in JEG-3 cells.** (A and B) Representative images and summary of migration and invasion assays in JEG-3/CD105 OE cells, JEG-3/shCD105 cells and their corresponding control cells. (C) Representative images and summary of apoptosis by flow cytometry. The values indicate the mean±SD of three independent experiments (*, P<0.05, Student's t-test). The images (A and B) are at ×40 magnification.

The results of the Transwell assays demonstrated that CD105 overexpression could promote the migration and invasion of JEG-3 cells *in vitro*, whereas knockdown was found to inhibit both processes (Fig. 3A and 3B). Flow cytometry showed that the rates of apoptosis in the JEG-3/CD105 OE and vector groups were 1.08 ± 0.26% and 3.44 ± 1.21%, respectively, whereas these rates were 26.27 ± 3.49% and 2.94 ± 0.51% in the JEG-3/shCD105 and vector groups, respectively (Fig 3C). Together, these data indicate that CD105 promotes JEG-3 cell migration and invasion and inhibits apoptosis* in vitro*.

### CD105 mediates drug-resistance in JEG-3 cells through the BMP9/Smad pathway

**Figure 4:**
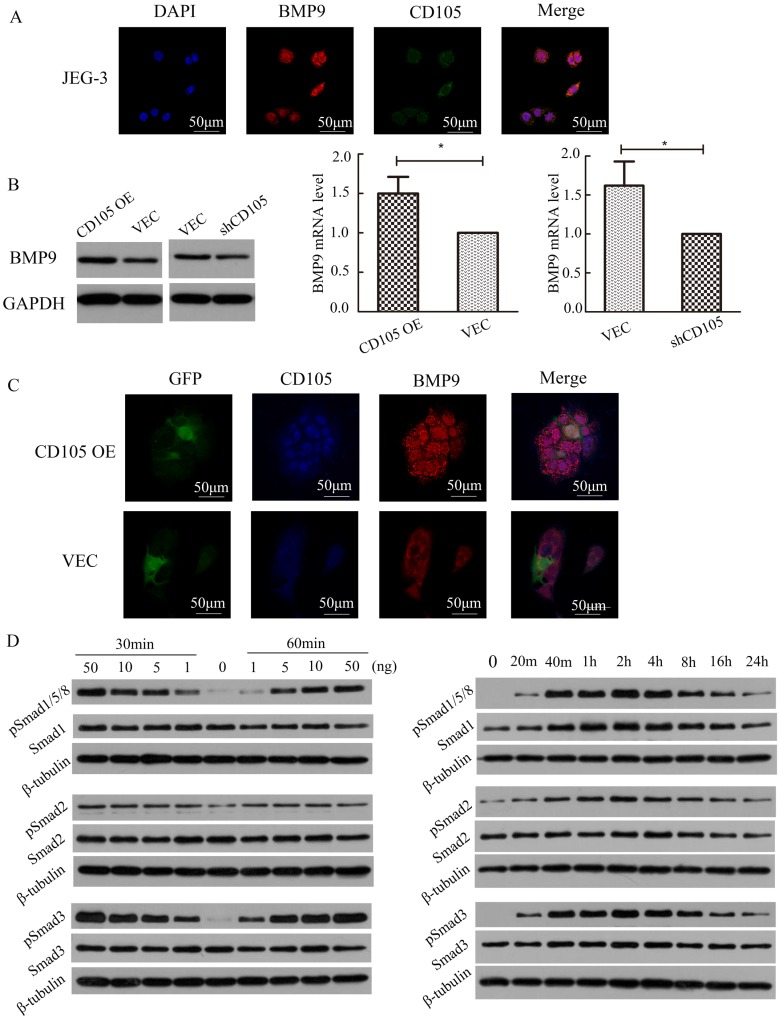
** CD105 mediates drug-resistance in JEG-3 cells through the BMP9/Smad pathway.** (A) The location of CD105 and BMP9 were determined in JEG-3 cells by immunofluorescence. (B) Western blotting and qRT-PCR showed that BMP9 was up-regulated in JEG-3/CD105 OE cells and down-regulated in JEG-3/shCD105 cells. (C) Immunofluorescence showed the fluorescence intensity of BMP9 was higher in JEG-3/CD105 OE cells compared to that in vector control cells. (D) Western blotting experiments indicated that BMP9 could induce Smad1/5/8, Smad2, and Smad3 phosphorylation in a dose- and time-dependent manner. The images (A and C) are at ×600 magnification.

IF showed that BMP9 also localizes to the cell membrane in JEG-3 cells and could co-localize with CD105 (Fig. 4A). To identify the relationship between BMP9 and CD105 in drug-resistant choriocarcinoma cells, we detected the cell viability of JEG-3/CD105 OE and JEG-3/shCD105 cells after BMP9 treatment. CCK8 assays showed that with increasing concentrations of BMP9, cell viability was gradually enhanced. At the same concentration of BMP9, the proliferation rate was highest in JEG-3/CD105 OE cells and lowest in JEG-3/shCD105 cells (Supplementary Fig. A). These results indicate that BMP9 can stimulate the proliferation of choriocarcinoma cells, and that CD105 overexpression enhances BMP9 induced proliferation. Next, we verified that BMP9 treatment could decrease drug sensitivity in choriocarcinoma cells (Supplementary Fig. B); this ligand was found to have a marked effect on JEG-3/CD105 OE cells, but only a slight influence on JEG-3/shCD105 cells. Altogether, these data indicate that BMP9 is involved in drug resistance in choriocarcinoma cells, and that CD105 might act as an essential co-receptor of BMP9.

To investigate the correlation between CD105 and BMP9, we determined the expression of BMP9 and CD105 in JEG-3/CD105 OE and JEG-3/shCD105 cells. Western blotting and qRT-PCR showed that BMP9 was up-regulated in JEG-3/CD105 OE cells and down-regulated in JEG-3/shCD105 cells (Fig. 4B). IF also showed that the fluorescence intensity of BMP9 was higher in JEG-3/CD105 OE cells compared to that in vector control cells (Fig. 4C). Together, these findings demonstrate a consistent relationship between the expression of CD105 and BMP9 in choriocarcinoma cells.

Activation of the Smad pathway is most critical for BMP9-induced signal transduction in endothelial cells 11. We therefore tested the ability of BMP9 to stimulate Smad activation in JEG-3 cells. Western blotting experiments indicated that BMP9 could induce Smad1/5/8, Smad2, and Smad3 phosphorylation in a dose- and time-dependent manner (Fig. 4D). Phosphorylation could be observed after 20 min of BMP9 treatment, was maximal at 2 h, and was sustained for at least 24 h. These results indicate that BMP9 activates Smad1/5/8, Smad2, and Smad3 in JEG-3 cells to regulate gene transcription and indicate that CD105 mediates drug resistance in choriocarcinoma cells through the BMP9/Smad pathway.

### CD105 and BMP9 are associated with poor prognosis in choriocarcinoma patients

**Figure 5:**
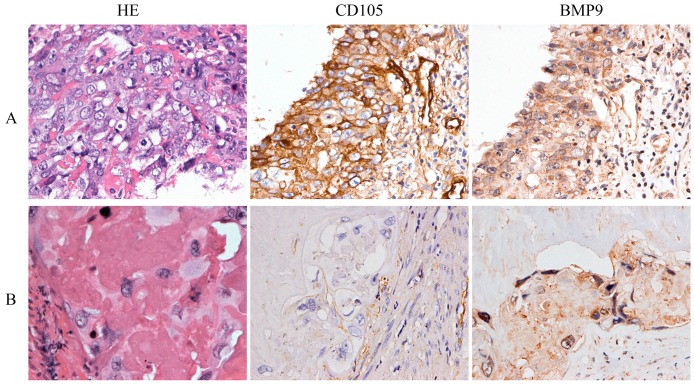
** CD105 and BMP9 are associated with poor prognosis in choriocarcinoma patients.** Representative images of HE staining and IHC with anti-CD105 and anti-BMP9 antibody. (A) High expressions of CD105 and BMP9 in tumor cells were observed in a woman who relapsed 6 months later after chemotherapy and hysterectomy. (B) Low expressions of CD105 and BMP9 in tumor cells were observed in a woman who experienced a durable remission later after chemotherapy and hysterectomy. The images (A and B) are at ×400 magnification.

The characteristics of 33 patients are listed in Table 1. During the follow-up time, eight cases relapsed after the combination of chemotherapy and surgery. HE staining showed that the average percentages of necrotic tissue, cytotrophoblasts, intermediate trophoblasts, and syncytiotrophoblasts were 88.27%, 5.76%, 3.26%, and 2.71%, respectively. IHC demonstrated that CD105 and BMP9 were mainly expressed in the cell membranes of tumor cells. Comparisons of patient clinicopathological characteristics and protein expression showed that CD105 and BMP9 expression levels were significantly associated with recurrence, but not correlated with age, β-hCG levels or different preoperative chemotherapy cycles (Supplementary Table S1-S2 and Fig. 5). In cytotrophoblasts and syncytiotrophoblasts, the expression of CD105 was significantly positively correlated with BMP9 expression (Supplementary Table S3). These results indicate that the expression levels of CD105 and BMP9 might be potential indicators to predict chemoresistance and poor prognosis.

## Discussion

Despite useful chemotherapeutic drugs and more effective combination treatments, drug resistance remains a major obstacle for the successful treatment of choriocarcinoma. In this study, we demonstrated that CD105 is up-regulated in drug-resistant choriocarcinoma cells.* In vitro*, overexpression of CD105 was found to decrease drug sensitivity, promote invasion and migration, and inhibit apoptosis in choriocarcinoma cells; accordingly, knockdown of CD105 had the opposite effects. Moreover, BMP9 and CD105 were consistently expressed both *in vivo* and *in vitro*, and CD105 was confirmed to mediate drug resistance in choriocarcinoma cells through the BMP9/Smad pathway. These findings provide a reference to study the mechanism underlying drug-resistant choriocarcinoma and suggest a possible new approach to reverse chemoresistance in choriocarcinoma patients.

CD105 is a well-known, reliable marker of endothelial cell proliferation, and is overexpressed in tumor neovasculature. However, increasing data suggest a broader role in tumor cell biology beyond endothelial expression alone. In renal and ovarian cancer research, CD105 expression was found to be associated with resistance to chemotherapy. Downregulation of CD105 significantly decreases tumorigenicity and gemcitabine resistance in renal cancer stem cell 12. Its inhibition could also decrease ovarian cancer cell viability, increase apoptosis, induce double-stranded DNA damage, and increase cisplatin sensitivity 13. An early study found that CD105 is strongly expressed on syncytiotrophoblasts throughout pregnancy and might be a cellular differentiation marker for choriocarcinoma cells 14. Results from Michael et al. also indicate that CD105 expression can be further enhanced by exposure to MTX 6, which is similar to our data.

Our results demonstrate that CD105 overexpression can influence the biological behaviours of choriocarcinoma cells, including decreasing drug sensitivity, promoting invasion and migration, and inhibiting apoptosis. However, CD105 has variable effects in other cancer types. In hepatocellular carcinoma cell lines, overexpression of this marker was found to promote cell invasion and migration 15. In esophageal squamous cell carcinoma, it is a tumor suppressor gene and overexpression was found to inhibit proliferation and invasion. The different effects of CD105 might be related to tumor heterogeneity and different signal pathways.

Several reports have suggested a role for BMP9 in both positively and negatively controlling cell biological behaviors in different cell types 8, 16, 17. In endothelial cells, BMP9 can interact with CD105 directly. Overexpression of CD105 increases the cascade reaction of the BMP9 pathway and this marker also regulates other ligand reactions through cellular and micro-environmental dependent mechanisms in endothelial cells 18. We demonstrated that BMP9 treatment could promote proliferation and decrease drug sensitivity in choriocarcinoma cells. However, these functions could not be obviously activated without CD105 expression. Thus, results indicate that BMP9 is involved in the drug resistance of choriocarcinoma cells, and that CD105 might act as an essential downstream factor of the BMP9 pathway. In addition, we determined that CD105 and BMP9 are positively correlated with each other. Therefore, the overexpression of CD105 might increase BMP9 pathway responses to induce drug resistance in choriocarcinoma cells. Whether there are other receptors or ligands involved in this activation process requires further study.

Upon binding to specific cell surface receptor kinases, BMP-mediated signal transduction begins with the phosphorylation of Smads and subsequent heterodimer formation. Traditionally, BMP9 was considered to activate Smad1/5/8 phosphorylation to produce biological effects 19. However in human pulmonary artery endothelial cells, BMP9 can also stimulate Smad2 activation 20, and can enhance TGFβ1-induced Smad2 and Smad3 phosphorylation 21. In vascular smooth muscle cells, BMP9 can markedly induce Smad1/5/8 phosphorylation, and marginally activate Smad2 and Smad3 22. Our data also suggest that BMP9 stimulates the phosphorylation of Smad1/5/8, Smad2, and Smad3. Whether the activating role of BMP9 in Smad2 and Smad3 signaling is direct or is dependent on enhanced TGFβ1 signaling requires further investigation. Together, the BMP9/Smad pathway is activated in choriocarcinoma cells and is suggested to play a critical role in drug resistance.

Although surgery is considered a less important approach for the management of choriocarcinoma, selected surgical procedures are necessary for removing chemoresistant lesions in the uterus and metastatic sites 23. However, chemoresistant lesions are associated with more necrotic tissues and fewer choriocarcinoma cells after chemotherapy; therefore, relevant studies regarding chemoresistant tissues are limited. Our study demonstrated that the expression of CD105 and BMP9 is positively correlated with each other in residual choriocarcinoma cells after chemotherapy, which is closely related to recurrence, but not related to age and β-hCG levels. Combining *in vivo* and* in vitro* results, the expression levels of CD105 and BMP9 might be important indicators to predict chemoresistance and poor prognosis. Further, combination analysis of the two factors might be more useful to identify the biological behaviours of choriocarcinoma.

Because of the rarity of CD105 expression in normal tissues, anti-CD105 therapy has the potential to offer tumor-directed therapy in addition to anti-angiogenic therapy. At present, several phase I and phase II, first-in human studies with TRC105 are ongoing for patients with refractory advanced or metastatic solid cancer 24-26. Our data strongly suggest that targeting CD105 is a potential important strategy for drug resistant choriocarcinoma and should be actively pursued as a modality for the treatment of this disease.

## Supplementary Material

Supplementary figures and tables.

## Figures and Tables

**Table 1:** Characteristics of 33 patients with choriocarcinoma

Characteristics	NO. (Ratio) or Median (Range)
Age	34.6 (24-53)
Preoperative β-hCG (mIU/mL)	122.6 (0-4554)
FIGO score	
≤6	3 (9.1%)
>6	30 (90.9%)
FIGO stage	
Ⅰ	7 (21.2%)
Ⅱ	1 (3.0%)
Ⅲ	23 (69.7%)
Ⅳ	2 (6.1%)
Chemotherapy cycles	8 (4-15)
Follow-up time (month)	24 (3-59)
Relapse	8 (24.2%)

## Abbreviations

β-hCG: β-humanchorionic gonadotropin
BMP-9: Bone morphogenetic protein-9
DMEM: Dulbecco's modified Eagle's medium
FBS: fetal bovine serum
FUDR: floxuridine
shRNA: small hairpin RNA
GFP: green fluorescent protein
MTX: methotrexate
qRT-PCR: quantitative real-time PCR
TGF-β: transforming growth factor-β

## References

[1] Ngu SF, Chan KK (2014). Management of Chemoresistant and Quiescent Gestational Trophoblastic Disease. Curr Obstet Gynecol Rep.

[2] Lee SR, Herbert IH, Michael KW (2010). A Phase 1 First-in-Human Study of TRC105 (Anti-Endoglin Antibody) in Patients with Advanced Cancer. Clin Cancer Res.

[3] Dolinsek T, Sersa G, Prosen L (2016). Electrotransfer of Plasmid DNA Encoding an Anti-Mouse Endoglin (CD105) shRNA to B16 Melanoma Tumors with Low and High Metastatic Potential Results in Pronounced Anti-Tumor Effects. Cancers (Basel).

[4] Martinez LM, Labovsky V, Calcagno ML (2015). CD105 expression on CD34-negative spindle-shaped stromal cells of primary tumor is an unfavorable prognostic marker in early breast cancer patients. PloS one.

[5] Saito T, Bokhove M, Croci R (2017). Structural Basis of the Human Endoglin-BMP9 Interaction: Insights into BMP Signaling and HHT1. Cell Rep.

[6] Worley MJ Jr, Elias KM, Horowitz NS (2018). Durable remission for a woman with refractory choriocarcinoma treated with anti-endoglin monoclonal antibody and bevacizumab: A case from the New England Trophoblastic Disease Center, Brigham and Women's Hospital and Dana-Farber Cancer Institute. Gynecol Oncol.

[7] Han B, Xiang Y, Sha GH (2010). Thymidylate synthase mRNA expression does not predict resistance to floxuridine in a choriocarcinoma cell line. J Reprod Med.

[8] Han B, Xiang Y, Wang Y (2010). Dihydrofolate reductase transcript level is not suitable for methotrexate-resistance prediction in choriocarcinoma cell line. Int J Gynecol Cancer.

[9] Herrera B, van Dinther M, Ten Dijke P (2009). Autocrine bone morphogenetic protein-9 signals through activin receptor-like kinase-2/Smad1/Smad4 to promote ovarian cancer cell proliferation. Cancer Res.

[10] Minhajat R, Li H, Kai K (2009). Anti-CD105 inhibits primary cancer growth and secondary hematogenous metastasis in a xenograft model. Vasc Dis Prev.

[11] Lamplot JD, Qin J, Nan G (2013). BMP9 signaling in stem cell differentiation and osteogenesis. Am J Stem Cells.

[12] Hu JH, Guan W, Liu PJ (2017). Endoglin Is Essential for the Maintenance of Self-Renewal and Chemoresistance in Renal Cancer Stem Cells. Stem cell reports.

[13] Ziebarth AJ, Nowsheen S, Steg AD (2013). Endoglin (CD105) contributes to platinum resistance and is a target for tumor-specific therapy in epithelial ovarian cancer. Clin Cancer Res.

[14] Letamendia A, Lastres P, Almendro N (1998). Endoglin, a component of the TGF-beta receptor system, is a differentiation marker of human choriocarcinoma cells. Int J Gynecol Cancer.

[15] Li Y, Zhai ZH, Liu D (2015). CD105 promotes hepatocarcinoma cell invasion and metastasis through VEGF. Tumor Biol.

[16] Herrera B, Dooley S, Breitkopf-Heinlein K (2014). Potential roles of bone morphogenetic protein (BMP)-9 in human liver diseases. International journal of molecular sciences.

[17] Ye L, Kynaston H, Jiang WG (2008). Bone morphogenetic protein-9 induces apoptosis in prostate cancer cells, the role of prostate apoptosis response-4. Mol Cancer Res.

[18] David L, Mallet C, Mazerbourg S (2007). Identification of BMP9 and BMP10 as functional activators of the orphan activin receptor-like kinase 1 (ALK1) in endothelial cells. Blood.

[19] Miyazono K (1999). Signal transduction by bone morphogenetic protein receptors: functional roles of Smad proteins. Bone.

[20] Upton PD, Davies RJ, Trembath RC (2009). Bone morphogenetic protein (BMP) and activin type II receptors balance BMP9 signals mediated by activin receptor-like kinase-1 in human pulmonary artery endothelial cells. J Biol Chem.

[21] Cunha SI, Pardali E, Thorikay M (2010). Genetic and pharmacological targeting of activin receptor-like kinase 1 impairs tumor growth and angiogenesis. J Exp Med.

[22] Zhu D, Mackenzie NC, Shanahan CM (2015). BMP-9 regulates the osteoblastic differentiation and calcification of vascular smooth muscle cells through an ALK1 mediated pathway. J Cell Mol Med.

[23] Wang X, Yang J, Li J (2017). Fertility-sparing uterine lesion resection for young women with gestational trophoblastic neoplasias: single institution experience. Oncotarget.

[24] Karzai FH, Apolo AB, Cao L (2015). A phase I study of TRC105 anti-endoglin (CD105) antibody in metastatic castration-resistant prostate cancer. BJU international.

[25] Gordon MS, Robert F, Matei D (2014). An open-label phase Ib dose-escalation study of TRC105 (anti-endoglin antibody) with bevacizumab in patients with advanced cancer. Clin Cancer Res.

[26] Apolo AB, Karzai FH, Trepel JB (2017). A Phase II Clinical Trial of TRC105 (Anti-Endoglin Antibody) in Adults With Advanced/Metastatic Urothelial Carcinoma. Clin Genitourin Cancer.

