# Supplementary material for: CD105 overexpression mediates drug-resistance in choriocarcinoma cells through BMP9/Smad pathway
Source: J Cancer. 2020 Jan 1;11(2):272–83. doi: 10.7150/jca.34965 (PMC6930438; doi:10.7150/jca.34965)
Supplement: Supplementary file 1 — Supplementary figures and tables. [file jcav11p0272s1.pdf]

Supplementary Table

Table S1

Association of clinicopathological parameters and expression of CD105 in 33 patients with choriocarcinoma (Mann-Whitney U test).

	Cytotrophoblasts		Intermediate trophoblasts		Syncytiotrophoblasts	
	n	*P*	n	*P*	n	*P*
Age						
≤35	19	0.912	22	0.206	20	0.977
>35	8		11		7	
Preoperative β-hCG (mIU/mL)						
≤5	18	0.260	24	0.977	17	0.671
>5	9		9		10	
Preoperative chemotherapy cycles						
≤4	14	0.135	19	0.343	13	0.166
>4	13		14		14	
Relapse						
NO	20	0.038	25	0.068	21	0.021
YES	7		8		6	

Table S2

Association of clinicopathological parameters and expression of BMP9 in 33 patients with choriocarcinoma (Mann-Whitney U test).

	Cytotrophoblasts		Intermediate trophoblasts		Syncytiotrophoblasts	
	n	*P*	n	*P*	n	*P*
Age						
≤35	19	0.774	22	0.373	20	0.588
>35	8		11		7	
Preoperative β-hCG (mIu/mL)						
≤5	18	0.176	24	0.231	17	0.141
>5	9		9		10	
Preoperative chemotherapy cycles						
≤4	14	0.790	19	0.527	13	0.728
>4	13		14		14	
Relapse						
NO	20	0.045	25	0.037	21	0.002
YES	7		8		6	

Table S3

Correlation between CD105 and BMP9 expression (Spearman test).

Cell type	*r*	*P*
Cytotrophoblasts	0.461	0.018
Intermediate trophoblasts	0.046	0.801
Syncytiotrophoblasts	0.531	0.004

Supplementary Figure legend

Supplementary Fig. (A) Cell viability by a CCK8 assay after treating with increasing concentrations of BMP9 in JEG-3/CD105 OE and JEG-3/shCD105 cells (*, $P<0.05$, Student's t-test). (B) Cell viability and IC_{50} values for MTX and FUDR with respect to JEG-3/CD105 OE and JEG-3/shCD105 cells after BMP9 treatment (10 ng/ml; *, $P<0.05$, Student's t-test).

A

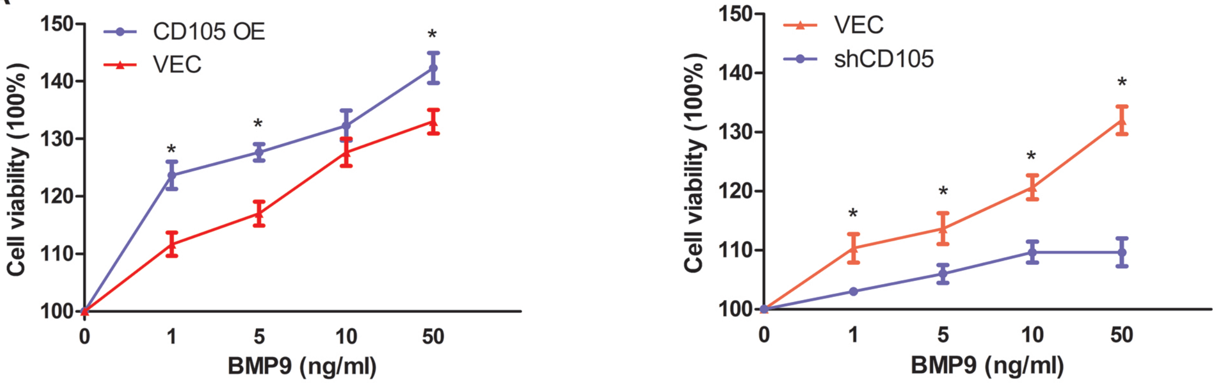

B

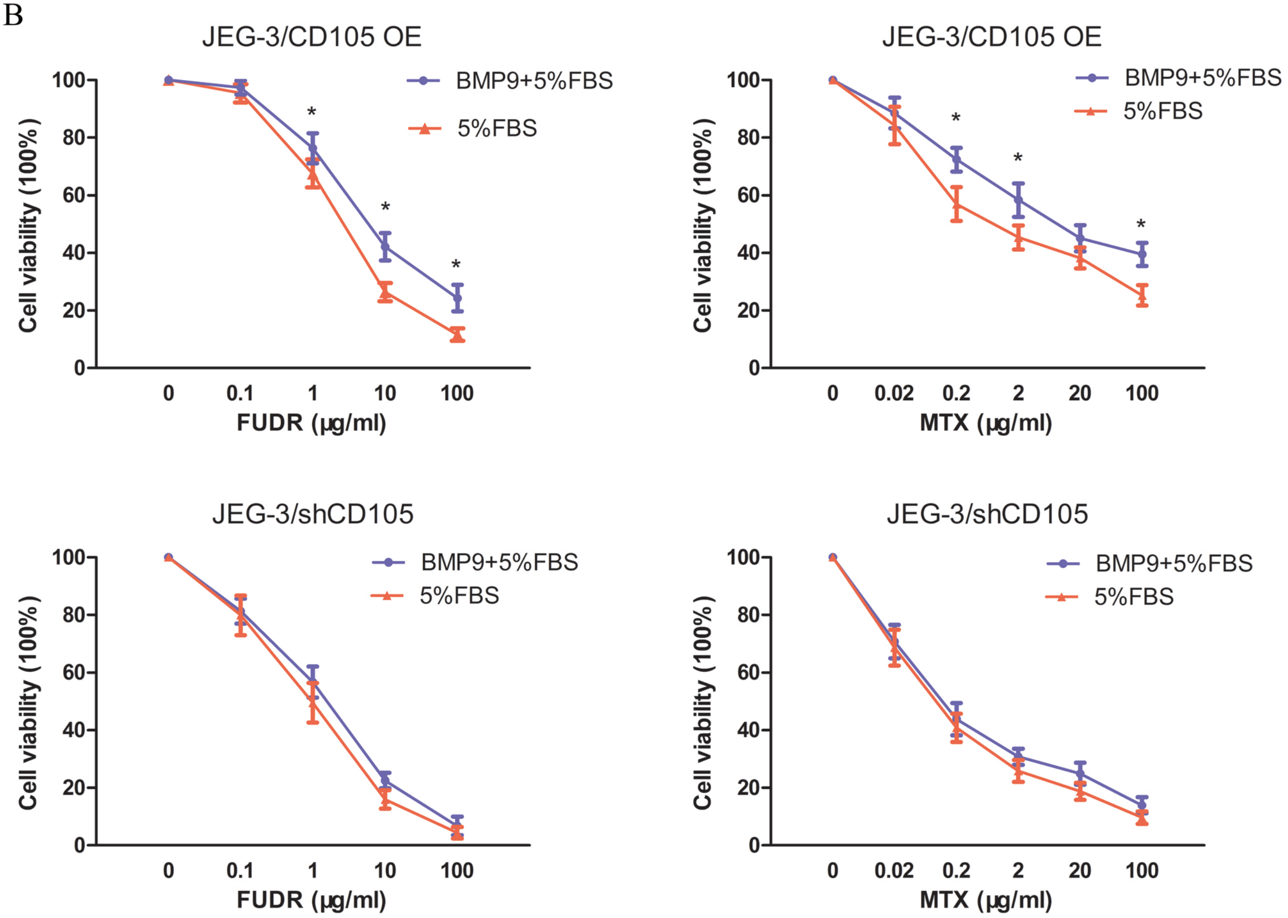

		CD105 OE		shCD105	
		FUDR	MTX	FUDR	MTX
IC50 μg/ml	BMP9+5%FBS	9.044	11.763	1.351	0.195
	5%FBS	3.242	1.793	0.883	0.122
